# Fields and Forests in Flames: Vegetation Smoke and Human Health

**DOI:** 10.1289/ehp.119-a386

**Published:** 2011-09-01

**Authors:** Bob Weinhold

**Affiliations:** Bob Weinhold, MA, has covered environmental health issues for numerous outlets since 1996. He is a member of the Society of Environmental Journalists.


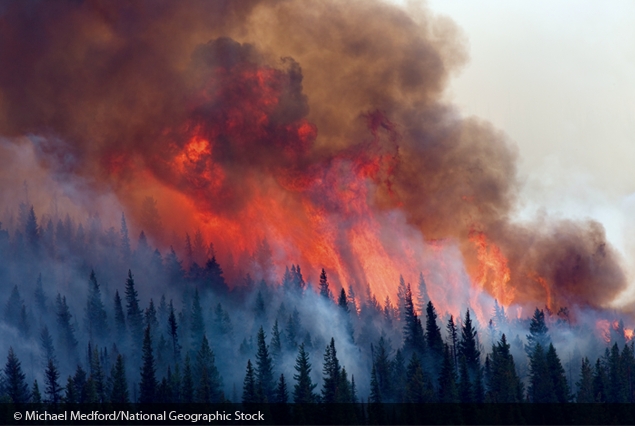
People have lived for tens of thousands of years in the presence of smoke from fires. That long period of adaptation tends to allow healthy younger adults in today’s environments to be generally resistant to serious adverse health effects from smoke from sources such as wildfires, prescribed forest burns, agricultural field burns, and peat bog fires, says Wayne Cascio, director of the U.S. Environmental Protection Agency (EPA) Environmental Public Health Division.

But a high percentage of people aren’t young, healthy adults. In the United States, nearly half the population suffers from at least one chronic illness,^1^ potentially placing them at risk for adverse effects from exposure to fire smoke. Children and older adults also are considered more vulnerable to smoke’s effects.^2^ The limited health research that’s been done on smoke from large-scale fires has provided some refinements to these general categories of vulnerable people, and new information occasionally emerges. There also has been a trickle of information identifying the toxic substances that characterize smoke from various kinds of fires, and pinning down the specific body systems that are vulnerable and the pathways through which damage occurs.

But much remains unknown about the varying toxicity of emissions from different types of vegetation fires and the vulnerability of specific groups of people, although a good deal of research has examined the adverse health effects of smoke related to heating and cooking with wood.^3^ Filling these voids is essential, Cascio says. “It is critically important to define who is at highest risk so that individual and community-based intervention strategies can be developed to specifically mitigate the health risks associated with smoke exposure,” he says. “The goal, of course, is to provide education or intervention to the most sensitive individuals in the most cost-effective way without needlessly worrying or interfering with the daily activities of [others].”

Such information can also help organizations and individuals who deal with fire threats as they work to integrate health concerns with many other factors, such as land management practices and programs, cultural mores, political influences, and funding.

## Conflagrations in the Woods

In the United States there has been an irregular but generally upward trend in the occurrence and severity of forest wildfires in the last 50 years. Each year between 1960 and 2010, some 1.1–9.9 million acres burned, with the highest acreage burned in 2006.^4^ At least 7 million acres burned in each of 7 of those 50 years; 6 such years occurred in the period 2000–2010.^4^ At least 5 million acres burned in each of 14 years, 10 of which fell in the period 1996–2010.

The annual acreage burned is expected to increase to about 10–12 million acres within just a few years.^5^ One of the forces expected to drive this projected increase in fires is climate change, which is expected to usher in increased drought, spreads in insect damage, and longer fire seasons, according to the U.S. Forest Service, Bureau of Land Management, U.S. Fish and Wildlife Service, National Park Service, Bureau of Indian Affairs, and National Association of State Foresters, and a growing body of independent studies.^5^^,^^6^^,^^7^ Among the areas expected to face the greatest increase in fire threats are the Southeast, Southwest, and West, although the Midwest and East also are expected to experience some increases.

However, some experts remain cautious, saying the science on wildfires and future impacts of climate change is still a work in progress. Brian Schwind, director of the U.S. Forest Service’s Remote Sensing Applications Center, says, “It’s a really complicated picture with a lot of variables. We’re early in the analytical phases. Sometimes we jump to conclusions a little fast.”

Historically, people have caused most wildfires. Of the 63,591–96,386 fires that occurred each year from 2001 to 2010, 80–90% were human-caused in any given year.^8^ For acreage burned, lightning often plays a much bigger role—when lightning fires strike backcountry areas, they are more often allowed to burn. But people still were the ignition source for 12–65% of the acreage burned in any of those years.^8^ Among the human causes of fires are arson, accidents, carelessness, and intentional prescribed fires designed to reduce acute threats or remove vegetation for planting, wildlife management, or other purposes.

**Figure:**
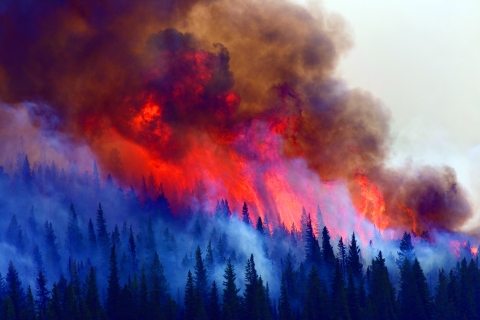
The Red Eagle Fire of 2006 burned more than 34,000 acres across Glacier National Park and adjacent Blackfeet Tribal Land. © Michael Melford/National Geographic Stock

More people have the opportunity to accidentally or intentionally start a fire as they increasingly move into the so-called wildland–urban interface, where residential areas butt up against and mingle with forests.^5^ That settlement pattern puts more people into close proximity to major fire sources, increasing the odds they’ll receive significant smoke exposures. It also results in an increase in man-made structures being burned by forest fires, says Stephen Mueller, a senior specialist in atmospheric science for the Tennessee Valley Authority. “Buildings and other structures usually contain plastic materials and various stored chemicals—pesticides, insecticides, paint, solvents, cleaning solutions, etc.—that release extremely toxic substances when burned,” he says. “This can represent a significant source of toxic air pollutants in certain areas.”

Globally, forest wildfire statistics are very scarce, says Pieter van Lierop, forestry officer with the Food and Agriculture Organization (FAO) of the United Nations. In 2010 hard data were available for less than half the world’s countries and only about three-fourths of the world’s forests.^9^ Inconsistent methods and reporting make it impossible to determine realistic total numbers of fires and acreage burned for any given year, or to detect trends. But it’s clear from global satellite images that significant fires in all types of vegetation occur multiple times every year on all continents except Antarctica.^10^ The percentage of these fires that are caused by humans is considered to be roughly 90–95%, van Lierop says.

Although hard global data aren’t available, researchers have used models and satellite images to calculate that fires in grasslands and savannas account for 44% of fire-derived carbon emissions, with 20% from tropical deforestation and degradation fires, 16% from tropical woodland fires, 15% from fires in forests outside the tropics, 3% from agricultural field burning, and 2% from peat fires.^11^ These estimates don’t necessarily reflect emissions of toxic substances, though, because emissions vary according to factors such as the type of vegetation burned, moisture content, fire temperature, wind conditions, how “aged” the smoke is, and time of year.

A global picture is also emerging for what are being termed “megafires,” according to a report sponsored by the FAO.^12^ The authors say the megafire label applies when a burn can’t be controlled by people without the help of natural forces such as rain, and it causes significant, long-lasting effects on an area’s environment and social and economic structure. Prime examples covered in some detail in the report include fires in Australia (2009), Botswana (2008), Brazil (1998), Greece (2007), Indonesia (1997/1998), Israel (2010), Russia (2010), and the United States (2003).

Other megafires have occurred in other years in some of these countries as well as in countries such as Canada, China, South Africa, Portugal, Spain, and Turkey. All were fueled in part by overzealous fire suppression or land practices that substantially altered the more fire-resistant natural vegetation mosaic and allowed fuels^13^ to accumulate.^12^ Drought and “extreme fire weather” (i.e., low humidity and high temperature combined with high winds) increased the hazard, and people almost always were the final straw, acting as the match in one way or another. Should these preventable fires increase as projected,^9^ their size and inability to be controlled will escalate the number of people exposed to toxic smoke and the length of time they are at risk.

## Who’s Affected by Wildfire Smoke?

The general health threat posed by smoke close to a fire has been widely recognized in the past decade by organizations such as the EPA,^14^ the U.S. Centers for Disease Control and Prevention,^2^ the California Department of Public Health,^15^ and the Pediatric Environmental Health Specialty Units, a network of academically based children’s environmental health experts.^16^ But people some distance away also are exposed. For instance, on many days in June 2011 the smoke plume from Arizona and New Mexico’s Wallow Fire extended as far as 1,000 miles.^17^

However, one of the large deficits in knowledge about the toxicity of smoke is the distance from a fire at which the smoke still poses a significant health threat, according to many experts. “Smoke changes as it travels, and the PM [particulate matter] might pose greater risk when it is closer to the source,” says Sarah Henderson, an environmental epidemiologist at the British Columbia Centre for Disease Control. However, she adds, “Anytime that smoke results in elevated PM, it has health effects.”

Smoke can contain thousands of individual compounds, in categories such as PM, hydrocarbons and other organic chemicals, nitrogen oxides, trace minerals, carbon monoxide, carbon dioxide, and water vapor.^15^ As just one example of elements in a complex mix, a 2009 fire in a mixed-evergreen forest in central Portugal generated emissions that included degradation products from biopolymers (such as levoglucosan from cellulose and methoxyphenols from lignin), *n*-alkanes, *n*-alkenes, *n*-alkanoic acids, *n*-alkanols, monosaccharide derivatives from cellulose, steroid and terpenoid biomarkers, polycyclic aromatic hydrocarbons (with retene being the most abundant), and even-carbon-number homologs of monoglycerides (which the authors say were identified for the first time as biomarkers in biomass burning aerosols).^18^

The health effects widely considered to be linked with wildfire smoke include exacerbation of preexisting respiratory conditions such as asthma and chronic obstructive pulmonary disease (COPD), reduced lung function, chest pain, and general symptoms such as eye irritation, fatigue, headache, dizziness, and stress.^15^ Woodsmoke exposure may depress the respiratory immune defenses^19^ and has been linked with emergency department visits for upper and lower respiratory effects.^20^ The evidence regarding cardiovascular effects has been mixed, but recent research is reinforcing these health issues as a possible area of concern, though sometimes only for certain categories of people in any given study.^21^^,^^22^^,^^23^^,^^24^^,^^25^^,^^26^

**Figure:**
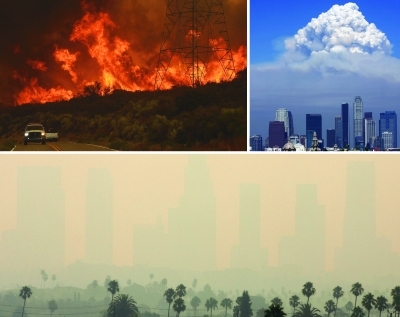
Clockwise from top left: The Station Fire burns north of Los Angeles, California, 30 August 2009. A wall of smoke from the fire rises over the city that same day; by the next day the Los Angeles skyline was obscured. The Station Fire was considered a “megafire,” meaning it could not be extinguished without the aid of natural forces such as rain. Clockwise from top left: © Gene Blevins/Reuters; © AP Photo/Jon Vidar; © Fred Prouser/Reuters

Based on the limited research conducted so far, public health officials generally consider children, older people, pregnant women, smokers, and people with chronic respiratory problems to be especially vulnerable to health effects from outdoor fires.^2^^,^^15^^,^^16^ Cascio says other populations that might be vulnerable and deserve greater study include diabetics, fetuses, people with cystic fibrosis and primary pulmonary hypertension, and those carrying certain genetic polymorphisms.

Refinements to this information are surfacing as studies trickle out. For instance, a study of bushfires in the Darwin, Australia, area in 2000, 2004, and 2005 found indigenous people were significantly more vulnerable to a range of respiratory disorders and had a statistically significant increase in hospital emissions for ischemic heart disease 3 days after initial exposure to smoke in relation to each 10-µg/m^3^ increase in PM_10_.^22^ The patients may have been at greater risk than others in the area because of greater underlying cardiorespiratory problems, the authors say.

This finding may be broadly applicable around the world. “Many other indigenous populations have a similar spectrum of social disadvantage and ill health as those from Australia, so the higher risk we saw in indigenous Australians is likely to be similar for those groups,” says Fay Johnston, lead author of the study and a public health physician and research fellow at the University of Tasmania’s Menzies Research Institute. This kind of knowledge can help refine local responses to fires. “If a severe smoke pollution event were to affect an indigenous community,” Johnston says, “the health outcomes are likely to be more serious, and public health officials would need to consider this when planning their responses.”

Another line of research involves the toxicologic differences between wildfire smoke and other types of particulate pollution. In an investigation of wildfires in central and northern California in 2008, researchers found that PM collected in the city of Tracy over 2 days at the peak of the fires was about 10 times more damaging to alveolar macrophages than ambient PM collected in the area under normal conditions, on an equal-dose basis.^27^ In California’s Central Valley, another team of researchers investigated differences between air in an urban area, Fresno, and near a wildfire about 100 miles to the northwest near Escalon.^28^ PM from each area induced very different inflammatory, oxidative stress, and xenobiotic responses in human bronchial epithelial cells, providing further evidence that it’s probably inappropriate to simply extrapolate findings on urban pollution to wildfire pollution.

However, urban air and wildfire smoke can have one thing in common—isocyanic acid, which was recently identified for the first time in outdoor air in each of these settings.^26^ The limited information available indicates the acid could plausibly contribute to cardiovascular problems and inflammation, although effects at the concentrations present in wildfire smoke have yet to be observed.

**Figure:**
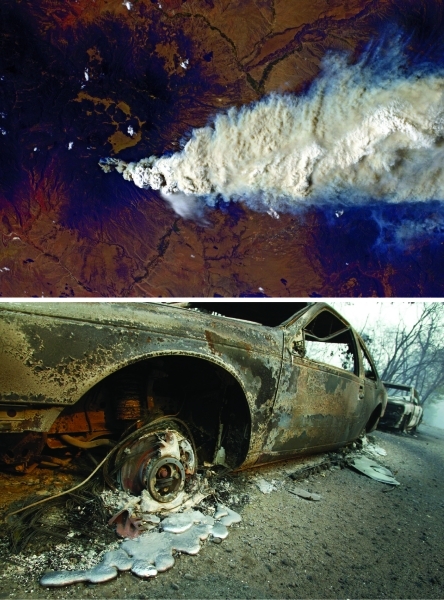
Top to bottom: The Las Conchas Fire burns in the Jemez Mountains of New Mexico southwest of Los Alamos National Laboratory, June 2011. If the fire had reached the nuclear waste stored at the laboratory, the result could have been a plume of radio­active smoke; extensive thinning around the facility reportedly averted such a disaster. October 2003 wildfires in San Diego County, California, destroyed more than 900 homes. The burning of buildings, vehicles, and other trappings of human society can add more toxic substances to wildfire smoke. op to bottom: NASA; © Mark Avery/Orange County Register/Corbis

Much more is generally known about the health risks posed by ground-level ozone, and a recent study indicates wildfires in the western United States can help spark the formation of the toxic substance, increasing ambient ozone by up to 50 ppb for a short period of time and potentially traveling long distances.^29^ Such bursts of ozone could cause affected areas to exceed the current federal 8-hour ozone standard of 75 ppb.^30^

In addition to polluting the air, wildfires can affect soil and water quality. In a study following fires in 2005 and 2006 in three watersheds in Southern California, researchers found organic or particulate-bound mercury in surface soils can be more readily deposited in waterways after a fire.^31^ Awareness of that tendency could lead to actions such as better testing of fish in affected waterways or improved sampling for water quality if the waterways are a drinking water source. However, it appears this phenomenon may depend on local soils, vegetation, waterways, and weather, because an analysis of 146 sites in Minnesota that had burned some time between 1759 and 2004 found intense fires had reduced soil mercury concentrations for tens, even hundreds, of years.^32^ In contrast, such reductions lasted only a year or so in the California settings.^31^

## Prescriptions for Fires

Wildfires are not the only large-scale fires humans encounter; in many areas around the world, people are exposed for substantial periods of time each year to smoke from prescribed (or controlled) fires, which are commonly used to preclude out-of-control wildfire threats. Experts attempt to do these on days with suitable weather (i.e., higher humidity, lower temperature, and low wind), when atmospheric conditions allow optimal smoke dispersion. They also try to restrict how the fire will spread, for instance by scraping out a perimeter line or setting fires from an outside boundary where terrain or winds will force the burn inward. But such fires still generate considerable smoke of varying compositions. In addition, they occasionally escape their intended boundaries and turn into wildfires.

**Figure:**
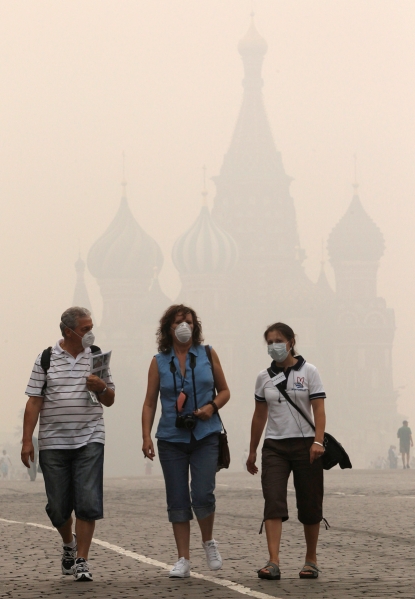
Moscow, Russia, during wildfires of the summer of 2010. Record-breaking high temperatures and drought conditions across Russia set the stage for these 2010 fires. These “extreme fire weather” factors are expected to occur more frequently in more locales in coming years. © Alexander Demianchuk/Reuters

U.S. federal, state, and other agencies have conducted prescribed burns on about 2.2 million acres per year in the past decade.^33^ Prescribed fires also are widely used globally, though hard data is scant.

Research on the health effects of prescribed burns is very limited. In a study of South Carolina prescribed fires, researchers found that plots in which the vegetation had been mechanically chipped in advance of burning emitted significantly less PM and carbon monoxide than nonchipped plots.^34^ The authors say this has implications for both firefighters and nearby communities. In Georgia, another team found emissions of most volatile organic compounds were much higher during the smoldering phase of prescribed fires in pine forests compared with the flaming phase.^35^ They also found emissions of several pinene compounds from prescribed fires were much higher than those from fireplace wood burning.

A study of prescribed burns in Arizona ponderosa pine forests found the emissions, which included PM, polycyclic aromatic hydrocarbons, organic carbon, elemental carbon, potassium, chlorine, sulfur, and silicon, were characteristic of smoldering, low-intensity burns.^36^ On the basis of the information in this and other studies, Marin Robinson, chairwoman of the Department of Chemistry and Biochemistry at Northern Arizona University, says, “I would argue that the biggest health effects associated with prescribed burns are short-term and involve susceptible individuals living in neighboring communities.”

Problems could be significant in some settings, though. In another study of the Darwin, Australia, area, researchers found that when PM_10_ from fires (many of which were prescribed burns) exceeded 40 µg/m^3^, emergency department admissions for asthma increased sharply.^37^ That concentration is far below the current 24-hour standard of 150 µg/m^3^ established by the U.S. EPA^38^ and even the level of 65–75 µg/m^3^ recommended in September 2010 by the agency’s Clean Air Scientific Advisory Committee.^39^ Other researchers report that smoke from prescribed fires in Australian bushlands contained acrolein, formaldehyde, and carbon monoxide at levels of concern.^40^

## Other Types of Fires: Bogs and Cropland

Although wildfires in peat bogs are the source of just a small fraction of the world’s smoke emissions, they can have a major impact on air quality in the areas where they burn. For instance, they were an important fuel in the megafires in Russia and Indonesia, and they occur widely in boreal forests. Since they become more flammable in normally moist areas that are undergoing extended drought, they could become an increasingly important smoke source if drought becomes more common in some areas.

A large June 2008 peat bog fire in North Carolina that burned about 6 weeks generated smoke affecting significant portions of the state. The fire, smoldering in peat 3–15 ft deep, had a poor oxygen supply and generated extensive smoke due to incomplete combustion. There were periods of PM_2.5_ concentration greater than 200 µg/m^3^ at ground-based monitors 200 km from the fire.^21^ The composition of peat fire emissions is known to differ substantially from forest fires, but the relative toxicity of these emissions is unknown. However, Mueller points out that low-temperature or smoldering combustion such as that associated with peat fires (and fireplaces) is notorious for emitting high amounts of carbon monoxide.

Whatever the specific toxic substances were, researchers studying cardiopulmonary-related emergency department visits associated with the 2008 peat bog fire found a 37% relative increase in heart failure (traits of the population studied, such as low income and high prevalence of health problems such as hypertension, diabetes, ischemic heart disease, and heart failure, may have contributed to susceptibility).^21^ They also reported increases in emergency department visits for COPD (73% increase), asthma (65% increase), and pneumonia and acute bronchitis (59% increase).^21^ Major peat fires were burning once again in North Carolina throughout late spring and summer of 2011.^41^^,^^42^^,^^43^

In agricultural fields, burning residue is a common practice worldwide. It’s done to kill pests, improve fertilization (by increasing nitrogen availability), and make planting easier, often at a lower cost than some other options such as mechanical tilling. As with forest wildfires, global data on field burning is limited. However, an analysis of satellite images from 2001 through 2003 indicated that about 1.5–1.6 million agricultural field burns occurred each year, accounting for an average 8–11% of annual global fire activity.^44^ Regions with the highest activity included the Russian Federation, Eastern Europe, and Central Asia.

**Figure:**
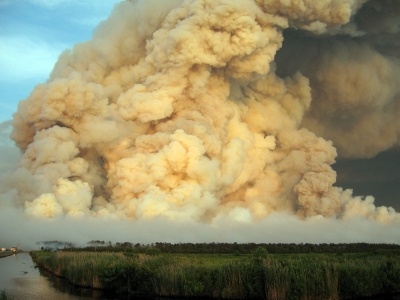
The Pains Bay peat fire in Dare County, North Carolina, was started 5 May 2011 by a lightning strike. The peat that feeds smoldering fires like this one can extend more than a dozen feet underground. These fires are notoriously hard to extinguish. North Carolina Forest Service

In the United States, field burning averaged 43% of the equivalent area burned by wildfires from 2003 to 2007 and peaked at 79% of the equivalent area in 2003.^45^ Field burning is a source of pollutants such as fine and coarse PM, nitrogen dioxide, sulfur dioxide, carbon monoxide, and methane.^46^ The states with the highest emissions (largely from sugarcane, wheat, rice, and bluegrass fields) are Arkansas, California, Florida, Idaho, Texas, and Washington. In those six states alone, about 15.5 million people live in “source” counties (that is, counties with crop burning areas), although it’s uncertain how many had significant smoke exposures.^46^ The percentage of a state’s population that lives in source counties can be quite high, such as 47% in Idaho and 25% in Arkansas.^46^

Field burning can occur for extended periods of time in any given area, leading to chronic exposures to the emissions.^46^ Smoke can readily waft beyond the source counties, although as with forest fires, the distance at which toxic effects occur remains largely unknown.

The limited research on health effects of field burning has found some significant respiratory and cardiopulmonary problems, says Jessica McCarty, a research scientist at Michigan Tech Research Institute. “The threat is highly variable, based on [local farming] laws, air quality laws, crop type, and cultural practices of burning,” she says.

## Few Studies, Many Possibilities

All together, there have been several dozen studies of health effects related to wildfires, prescribed forest burns, peat bog fires, and agricultural field burning. That’s a relatively small number given the huge variation in source material that can burn, the various underlying conditions of people who can be affected, and other variables (by comparison, more than 1,700 health studies have been conducted for ground-level ozone). One reason for that dearth is that the research is hard to do.

**Figure:**
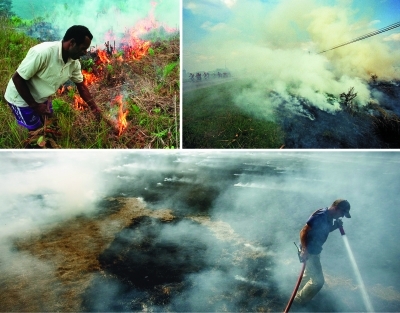
Clockwise from top left: a sweet potato field in Indonesia; a sugarcane field in Cuba; a bluegrass field in Rathdrum, Idaho. Agricultural field burning is practiced around the world as a relatively inexpensive way to prepare fields for crops. Some jurisdictions around the world require permits prior to an agricultural burn, addressing issues such as extent and timing, in an effort to reduce health risks to area residents. Clockwise from top left: © Rob Huibers/Panos Pictures; © Chris de Bode/Panos Pictures; © Jerome A. Pollos/AP Photo

Johnston explains that fires often are short-term events, and appropriate individual health data are often lacking, as are data on possible confounders. Sometimes the available study population isn’t large enough to generate clear associations. Another major limitation is the lack of monitoring data in burn areas. More recent studies are beginning to circumvent this issue by using tools such as pollution models and satellite data. But those approaches still have limitations that often don’t allow them the precision of ground monitors.

Despite the difficulties, “it is clear that more research must be done to fully characterize the chemical composition of the particulate matter arising from these various sources,” Cascio says. Ralph Delfino, vice chair for research and graduate studies at the University of California, Irvine, Department of Epidemiology, says more information is needed about the mechanisms through which fire emissions cause harm. “It would also be useful from a public health perspective to have better information for health advisories such as data to forecast the locations of smoke plumes and data on the clinical characteristics of potentially susceptible populations to enable targeted alerts. There is sufficient evidence to warn people with persistent asthma who may benefit from the use of preventive antiinflammatory medications,” he says. He adds that improved application of satellite imagery plus ground-level air monitoring could help in forecasting smoke movements.

Despite the potential public health benefits to be had from these types of studies, Delfino says he has repeatedly found little support for this kind of research, possibly because decision makers and funders are rarely exposed to significant smoke. “People change their minds when they are in the middle of it, though,” he says.
